# Supplementary material for: Curing Kinetics of Methylene Diphenyl Diisocyanate—Based Polyurethane Elastomers
Source: Polymers (Basel). 2022 Aug 27;14(17):3525. doi: 10.3390/polym14173525 (PMC9459966; doi:10.3390/polym14173525)
Supplement: Supplementary file 1 [file polymers-14-03525-s001.zip › polymers-1868442-supplementary.pdf]

The reaction temperature is 81 ℃, and the calculation results are shown in Table S1:

Table S1. Calculation of α-t relationship by Equations (17) and (18).

α	$\frac{d\alpha}{dt}$	*t*/min
0.05	0.06005	0.01388
0.08	0.06615	0.02016
0.1	0.06827	0.02441
0.15	0.06971	0.03586
0.2	0.0678	0.04916
0.25	0.06394	0.06516
0.3	0.05889	0.0849
0.35	0.05312	0.10981
0.4	0.04698	0.1419
0.45	0.04072	0.1842
0.46	0.03947	0.19426
0.48	0.03698	0.21634
0.49	0.03575	0.22847
0.5	0.00977	0.85275
0.55	0.01008	0.90912
0.6	0.01016	0.98413
0.65	0.00999	1.08462
0.7	0.00955	1.22225
0.75	0.00881	1.41816
0.8	0.00778	1.71458
0.85	0.00641	2.20956
0.9	0.0047	3.19449
0.95	0.00259	6.10339
0.98	0.00112	14.5943
0.99	5.81566×10^{-4}	28.37168
